# Assessing the Performance of ChatGPT in Medical Biochemistry Using Clinical Case Vignettes: Observational Study

**DOI:** 10.2196/47191

**Published:** 2023-11-07

**Authors:** Krishna Mohan Surapaneni

**Affiliations:** 1 Panimalar Medical College Hospital & Research Institute Chennai India

**Keywords:** ChatGPT, artificial intelligence, medical education, medical Biochemistry, biochemistry, chatbot, case study, case scenario, medical exam, medical examination, computer generated

## Abstract

**Background:**

ChatGPT has gained global attention recently owing to its high performance in generating a wide range of information and retrieving any kind of data instantaneously. ChatGPT has also been tested for the United States Medical Licensing Examination (USMLE) and has successfully cleared it. Thus, its usability in medical education is now one of the key discussions worldwide.

**Objective:**

The objective of this study is to evaluate the performance of ChatGPT in medical biochemistry using clinical case vignettes.

**Methods:**

The performance of ChatGPT was evaluated in medical biochemistry using 10 clinical case vignettes. Clinical case vignettes were randomly selected and inputted in ChatGPT along with the response options. We tested the responses for each clinical case twice. The answers generated by ChatGPT were saved and checked using our reference material.

**Results:**

ChatGPT generated correct answers for 4 questions on the first attempt. For the other cases, there were differences in responses generated by ChatGPT in the first and second attempts. In the second attempt, ChatGPT provided correct answers for 6 questions and incorrect answers for 4 questions out of the 10 cases that were used. But, to our surprise, for case 3, different answers were obtained with multiple attempts. We believe this to have happened owing to the complexity of the case, which involved addressing various critical medical aspects related to amino acid metabolism in a balanced approach.

**Conclusions:**

According to the findings of our study, ChatGPT may not be considered an accurate information provider for application in medical education to improve learning and assessment. However, our study was limited by a small sample size (10 clinical case vignettes) and the use of the publicly available version of ChatGPT (version 3.5). Although artificial intelligence (AI) has the capability to transform medical education, we emphasize the validation of such data produced by such AI systems for correctness and dependability before it could be implemented in practice.

## Introduction

A new powerful artificial intelligence (AI)–driven large language model called “ChatGPT” has gained increasing attention. Within 3 months of its launch, ChatGPT has attracted over millions of users with its ability to generate astounding and diverse conversations based on enormous amounts of data, and achieve milestones by performing well on competitive medical examinations [1,2]. This impressive conversational chatbot was developed by OpenAI (San Francisco, California) on November 30, 2022, and is currently funded by Microsoft and others [3], having significantly impacted the field of education. However, there are conflicting reactions among educators globally regarding ChatGPT’s amazing capacity to perform difficult tasks in education because this development in AI appears to completely transform current educational practices [4].

In the medical science context, ChatGPT is believed to be able to reshape medical education, research, and clinical decision management by rapidly creating content to learn, providing quick access to information, and creating a personalized learning experiences [5]. Recently, ChatGPT had also cleared the United States Medical Licensing Examination (USMLE) with an acceptable score, thus reinforcing the usability of such AI models to enhance medical education [6,7]. However, literature about the performance of ChatGPT in biochemistry and its ability to interpret clinical conditions and provide valuable contributions to medical education is lacking. Therefore, we aimed to assess the diagnostic and interpretation ability of ChatGPT using clinical case vignettes in medical biochemistry.

## Methods

ChatGPT’s performance was evaluated in clinical biochemistry using 10 clinical case vignettes. We used ChatGPT’s version 3.5 without the Plus subscription. The 10 clinical case vignettes in medical biochemistry were randomly selected from *Biochemistry and Genetics PreTestTM Self-Assessment and Review, Third Edition* [8], wherein the correct answers and subsequent explanations are also available; this was used as the reference material [8] to evaluate ChatGPT-generated answers. All clinical case vignettes were in the format of clinical case–based multiple-choice questions and were chosen from chapters on carbohydrate metabolism, lipid metabolism, amino acid metabolism, heme metabolism, and acid-based equilibria. All vignettes were typed exactly with the same options per our reference material [8] in ChatGPT’s input field. ChatGPT-generated responses were saved and documented. The reference material [8] was used to check ChatGPT-generated answers and explanations. For all 10 clinical cases, ChatGPT chose 1 option from the multiple choices and provided an explanation for the answers. The correctness of ChatGPT-generated answers was checked using the answers and explanation as provided in the reference material [8] by 2 expert faculty members (with postgraduate qualifications and considerable teaching experience in medical biochemistry) independently to avoid bias. All the answers provided in the reference material [8] were cross-referenced with the standard biochemistry textbooks including Harper's Illustrated Biochemistry (31st edition) [9] and *Lippincott Illustrated Reviews: Biochemistry* [10]. All vignettes used for this were numbered 1 through 10. All the answers were rechecked twice by typing the same question and regenerating the responses. However, while conducting this study, ChatGPT was not informed about the incorrect responses it had generated, although it is considered standard practice to provide an opportunity to chatbots to acknowledge its errors. ChatGPT was only used to obtain the responses for the clinical case vignettes; it was not used to write any part of the manuscript.

## Results

The weightage of clinical cases is shown in Table 1.

**Table 1:** Weightage of clinical cases (N=10).

Chapter	Weightage, %	Case numbers
Carbohydrate metabolism	20	1 and 6
Lipid metabolism	30	4, 8, and 9
Amino acid metabolism	20	3 and 7
Heme metabolism	10	10
Acid-base equilibria	20	3 and 5

In the first attempt, upon evaluating the answers using our reference material [8], out of the 10, ChatGPT provided the correct answers for 4 questions and incorrect answers for 6 questions. ChatGPT-generated answers matched our answer key for 4 questions (cases 4, 6, 7, and 10), and the explanation provided was also in accordance with the one provided in our reference material [8]. There were discrepancies between ChatGPT-generated answers and original answer keys for 6 questions (cases 1, 2, 3, 5, 8, and 9). In the second attempt, ChatGPT provided correct answers for 6 questions and incorrect answers for 4 questions out of the 10 cases used. Questions for which a correct answer was generated in the first attempt had the same correct answer in the second attempt (cases 4, 6, 7, and 10). Answers to the other 6 questions—for which ChatGPT generated incorrect answers in the first attempt—were changed, and in the second attempt, correct answers were generated for 2 questions in accordance with our reference material (cases 5 and 9) [8]. Three of the questions answered incorrectly in the first attempt again had the same incorrect answers in the second attempt (cases 1, 2, and 8). Surprisingly, in 1 case (case 3), multiple answers were obtained on each attempt. This could be attributed to the complexity of the case scenario, stemming from the need to address multiple critical medical facets about amino acid metabolism; this case required a delicate balance of clinical knowledge, surgical expertise, understanding of neonatal nutrition, and awareness of amino acid essentiality to ensure the best treatment outcome. The clinical cases used are summarized in Textbox 1, and the answers in our reference material [8] and ChatGPT-generated answers are presented in Table 2. The results of this study are presented with image answers generated by ChatGPT in the first attempt (Multimedia Appendices 1-10). Discrepancies in answers with different answers provided in multiple attempts in case 3 are presented in Multimedia Appendices 11-14.

**Clinical case vignettes used in this study (extracted from Biochemistry and Genetics PreTestTM Self-Assessment and Review, Third Edition, 2007) [8]. (Case descriptions have been quoted as text inputted in and responses generated by ChatGPT and are hence unaltered.)**

**Clinical case 1**

A teenager is brought in by his parents after his physical education teacher gives him a failing grade. The teacher has scolded him for malingering because he drops out of activities after a few minutes of exercise complaining of leg cramps and fatigue. A stress test is arranged with sampling of blood metabolites and monitoring of exercise performance which of the following results after exercise would support diagnosis of glycogen storage disease in this teenager?

- Increased oxalate, decreased glucose
- Increased glycerol and glucose
- Increased lactate and glucose
- Increased pyruvate and stable glucose
- Stable lactate and glucose

**Clinical case 2**

A male infant does well in the nursery but seems to have a reaction to serial introduced at age 6 weeks the infant begins vomiting severely often spewing vomitus across the crib (projectile vomiting). Concern about food allergy persists until an experienced surgeon sits with her hand over the infant stomach for 20 minutes at the bedside, feeling a small oval shape that has been described as an olive. The surgeon obtains electrolytes and blood gases preparatory to anaesthesia which of the combinations of laboratory results below and their interpretation are most likely for this infant?

- Low Pco2, normal bicarbonate, normal chloride, high pH – pure respiratory alkalosis
- Low Pco2, low bicarbonate, low pH, low chloride – compensated metabolic acidosis
- Normal Pco2, low bicarbonate, low pH, normal chloride – pure metabolic acidosis
- High Pco2, normal bicarbonate, low pH, normal chloride – pure respiratory acidosis
- Normal Pco2, high bicarbonate, high pH, low chloride- pure metabolic alkalosis

**Clinical case 3**

A newborn with meconium ileus (plugging of the small intestine with meconium or fetal stool) is found to have air in the bowel wall (pneumatosis intestinalis) and free air in the abdomen. Antibiotics are begun for suspected peritonitis and emergency surgery is performed to remove the diseased intestinal segment and heal the intestinal perforation that led to air in the abdomen. Because the gut must be kept at rest for healing meconium peritonitis was usually fatal until parental alimentation solutions were developed. Hyperalimentation consists of essential amino acids and other metabolites that provide a positive calorie balance while keeping the bowel at rest. The alimentation solution must be kept to a minimum of metabolites because of its high osmotic load that necessitates frequent changing of intravenous sites catheterization of a large vein. Which of the following amino acids could be excluded from the alimentation solution?

- Cysteine
- Phenylalanine
- Histidine
- Methionine
- Tryptophan

**Clinical case 4**

A 2-year-old girl has been healthy until the past weekend when she contracted a viral illness at day care with vomiting, diarrhea and progressive lethargy. She presents to the office on Monday with disorientation, a barely rousable sensorium, cracked lips, sunken eyes, lack of tears, flaccid skin with “tenting” on pinching, weak pulse with low blood pressure and increased deep tendon reflexes. Laboratory tests show low blood glucose, normal electrolytes, elevated liver enzymes and (on chest X ray) a dilated heart. Urinalysis reveals no infection and no ketones. The child is hospitalised and stabilised with 10% glucose infusion and certain admission laboratories come back 1 week later showing elevated medium chain fatty acyl carnitines in blood and 6 to 8 carbon di carboxylic acids in the urine the most likely disorder in this child involves which of the following?

- Defect of medium chain coenzyme a dehydrogenase
- Defect of medium chain fatty acid synthetase
- Mitochondrial defect in the electron transport chain
- Mitochondrial defect in fatty acid transport
- Carnitine deficiency

**Clinical case 5**

A 2-day-old neonate becomes lethargic and uninterested in breastfeeding. Physical examination reveals hypotonia (low muscle tone), muscle twitching that suggests seizures and tachypnea (rapid breathing). The child has a normal heart beat and breath sounds with no indication of cardio respiratory disease. Initial blood chemistry values include normal glucose, sodium, potassium, chloride and bicarbonate (HCO3-) levels; initial blood gas values reveal a pH of 7.53, partial pressure of oxygen (PO2) normal at 103 mmHg and partial pressure of carbon dioxide (PCO2) decreased at 27 mmHg. Which of the following treatment strategies is most appropriate?

- Administer alkali to treat metabolic acidosis
- Administer alkali to treat respiratory acidosis
- Decrease the respiratory rate to treat metabolic acidosis
- Decrease the respiratory rate to treat respiratory alkalosis
- Administer acid to treat metabolic alkalosis

**Clinical case 6**

After a term uncomplicated gestation, normal delivery, and unremarkable nursery stay, a 10 day old female is readmitted to the hospital because of poor feeding, weight loss, and rapid heart rate. Antibiotics are started as a precaution against sepsis, and initial testing indicates an unusual echo cardiogram with a very short PR interval and a large heart on X ray. initial concern about a cardiac arrhythmia changes when a large tongue is noted, causing concern about glycogen storage disease type 2 (Pompe disease-232300-table3). Which of the following best explains why Pompe disease is more severe and lethal compared to other glycogen storage diseases?

- The deficiency is a degradative rather than synthetic enzyme
- The deficiency involves a liver enzyme
- The deficiency involves a lysosomal enzyme
- The deficiency causes associated neutropenia
- The deficiency involves a serum enzyme

**Clinical case 7**

An adolescent female develops hemiballismus (repetitive throwing motion of the arms )after anesthesia for a routine operation. She is tall and lanky and it is noted that she and her sister both had previous operations for dislocated lenses of the eyes. The symptoms are suspicious for the disease homocystinuria (236300). Which of the following statements is descriptive of this disease?

- Patients may be treated with dietary supplements of vitamin B 12
- Patients may be treated with dietary supplements of vitamin C
- There is deficient excretion of homocysteine
- There is increased excretion of cysteine
- There is a defect in the ability to form cystathionine from homocysteine and serine

**Clinical case 8**

Children with very long or long chain fatty acid oxidation disorders are severely affected from birth, while those with short or medium chain oxidation defects may be asymptomatic until they have an intercurrent illness that causes prolonged fasting. the severe symptoms of longer chain diseases are best explained by which of the following statements?

- Longer chain fatty acids inhibit gluconeogenesis and deplete serum glucose needed for brain metabolism
- Glycogen is the main fuel reserve of the body but is quickly depleted with fasting
- Starch is an important source of glucose and is inhibited by high fatty acid concentration
- Triacylglycerol are the main fuel reserve of the body and are needed for energy production in actively metabolising tissues
- Longer chain fatty acids form micelles and blocked synapsis

**Clinical case 9**

A 45-year-old man is found to have an elevated serum cholesterol of 300 mg percent measured by standard conditions after a 12-hour fast. Which of the following lipoproteins would contribute to a measurement of plasma cholesterol in a normal person following a 12 hour fast?

- Very-low-density lipoprotein (VLDL) and low-density lipoproteins (LDL)
- High-density lipoproteins (HDL) and low-density lipoproteins (LDL)
- Chylomicrons and very-low-density lipoproteins (VLDL)
- Chylomicron remnants and very-low-density lipoproteins (VLDL)
- Low-density lipoproteins (LDL) and adipocyte lipid droplets

**Clinical case 10**

35 year-old-man presents to the emergency room with an acute abdomen (severe abdominal pain with tightness of muscles, decreased bowel sounds and vomiting and/or diarrhea). He has been drinking, and a urine sample is unusual because it has a port-wine colour. past history indicates several prior evaluations for abdominal pain, including and appendectomy. The physician notes unusual neurological symptoms with partial paralysis of his arms and legs. at first concerned about food poisons like Botulism, the physician recalls that acute intermittent porphyria may cause these symptoms (176000) and consult a gastroenterologist. Elevation of which of the following urinary metabolites would support a diagnosis of porphyria?

- Urobilinogen and bilirubin
- Delta-aminolevulinic acid and porphobilinogen
- Biliverdin and stercobilin
- Urobilin and urobilinogen
- Delta-aminolevulinic acid and urobilinogen

**Table 2:** ChatGPT’s performance in medical biochemistry using clinical case vignettes (N=10).

Clinical case number	Answer in reference material^a^	Answer generated by ChatGPT		Correctness of the answer generated by ChatGPT
		First attempt	Second attempt	
1	E	C	C	Incorrect
2	E	D	D	Incorrect
3	A	B	Second attempt: C; third attempt: E; fourth attempt: none	Different answers in multiple attempts
4	A	A	A	Correct
5	D	None	D	First attempt: incorrect; second attempt: correct
6	C	C	C	Correct
7	E	E	E	Correct
8	D	A	A	Incorrect
9	B	A	B	First attempt: incorrect; second attempt: correct
10	B	B	B	Correct

^a^Response options indicated as A through E.

## Discussion

Our evaluation of ChatGPT’s performance in medical biochemistry yielded average results. ChatGPT’s performance cannot be regarded as high owing to numerous discrepancies between ChatGPT-generated answers and the original answer key [8]. Also, the difference between ChatGPT-chosen options in the first and subsequent attempts indicates that as the complexity of the content increased, the precision of the generated answers decreased, emphasizing the need to verify the answers generated by this chatbot before its implementation. Hence, validating the information generated is crucial before we can completely rely on such AI-powered tools.

Large language models such as ChatGPT may enhance student engagement and learning by assisting in web-based learning by generating pertinent and comprehensive content [11]. Assessment of ChatGPT’s knowledge of microbiology in competency-based medical education provided impressive results with an 80% accuracy rate in answering first-order and second-order knowledge questions [12]. ChatGPT also performed well in diagnosing and interpreting a case scenario in clinical toxicology. However, medicine functions beyond the capacity to provide a correct diagnosis and relevant information. ChatGPT cannot replace the human ability of eliciting history and take prompt actions [13].

ChatGPT’s acceptance as an effective learning tool in medical education is still a debate. On comparing the knowledge and interpretation skills of medical students and ChatGPT in a parasitology examination, the correctness of answers and acceptability of explanations were lower for ChatGPT-generated responses than for medical students’ answers [14]. In the context of the development of medical education curricula, the performance of ChatGPT in outlining content for sessions on lipid metabolism and generating learning objectives and evaluation questions was not highly commendable, indicating the need to verify the information and beware of misleading or incorrect information that could be possibly generated by these AI tools [15].

Thus, diversity in ChatGPT’s performance in various medical sciences is a major limitation for AI to be accepted as a productive learning platform for students and educators and to be successfully used to reframe medical education and research [16]. But, ChatGPT is certainly a highly beneficial asset that can be used to achieve several milestones if used with caution and proper authentication [17]. Thus, more studies should focus on testing ChatGPT in various fields of medicine to assess its performance and frame appropriate regulations in the implementation of AI-based systems in medical education and research.

This study has certain limitations. First, only 10 clinical case vignettes were used to assess ChatGPT’s potential in solving them. Owing to the smaller sample size, more detailed studies would be required to confirm and disseminate the findings of this study. Further, only the publicly available version of ChatGPT (version 3.5) was used. Thus, ChatGPT’s performance and the quality of responses are limited to the scope of this version.

This study analyzed the performance of ChatGPT in medical biochemistry using clinical case vignettes. From the results of this study, it is certain that before we use the content generated by AI innovations such as ChatGPT, it is important to assess the reliability and accuracy of the information provided. As huge amounts of data are being handled by AI tools, misinformation or disinformation are the most common issues encountered. However, ChatGPT undoubtedly has a high potential to enhance teaching, learning, and assessment strategies in the field of medical education. Although AI cannot replace humans, chatbots such as ChatGPT have good prospects for advancing medical education under expert surveillance. As this is a rapidly advancing field, newer and upgraded versions can be expected to be released with higher accuracy and with minimal errata. Hence, the scope of future research should be widened with the aim of approving AI-generated content with validity and reliability. Once this is achieved, ChatGPT will have the potential to emerge as the most rapid and efficient information-generating tool that can certainly transform the medical education system.

## Appendix

Multimedia Appendix 1 Case 1 ChatGPT performance.

Multimedia Appendix 2 Case 2ChatGPT performance.

Multimedia Appendix 3 Case 3 ChatGPT performance.

Multimedia Appendix 4 Case 4 ChatGPT performance.

Multimedia Appendix 5 Case 5 ChatGPT performance.

Multimedia Appendix 6 Case 6 ChatGPT performance.

Multimedia Appendix 7 Case 7 ChatGPT performance.

Multimedia Appendix 8 Case 8 ChatGPT performance.

Multimedia Appendix 9 Case 9 ChatGPT performance.

Multimedia Appendix 10 Case 10 ChatGPT performance.

Multimedia Appendix 11 Case 3 ChatGPT performance – 2nd attempt.

Multimedia Appendix 12 Case 3 ChatGPT performance– 3rd attempt.

Multimedia Appendix 13 Case 3 ChatGPT performance – 4th attempt.

Multimedia Appendix 14 Case 3 ChatGPT performance – 4th attempt (Contd...).

## Abbreviations

AI: artificial intelligence
USMLE: United States Medical Licensing Examination

## Data Availability

The data that support this study are available upon request from the corresponding author.
