# Supplementary figures and images for: Assessing the Performance of ChatGPT in Medical Biochemistry Using Clinical Case Vignettes: Observational Study
Source: JMIR Med Educ. 2023 Nov 7;9:e47191. doi: 10.2196/47191 (PMC10664016; doi:10.2196/47191)

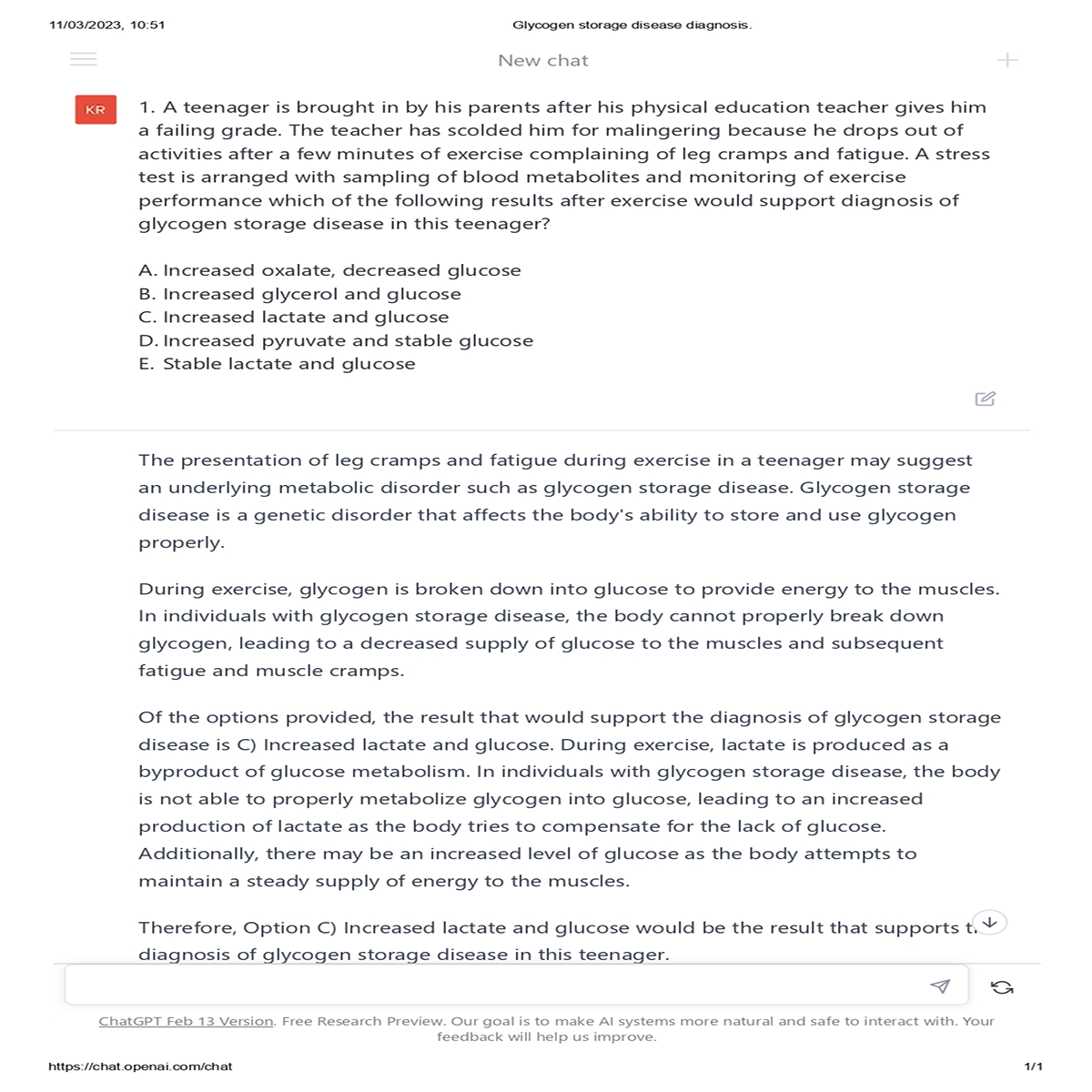

Supplement: Multimedia Appendix 1 [file mededu_v9i1e47191_app1.png]

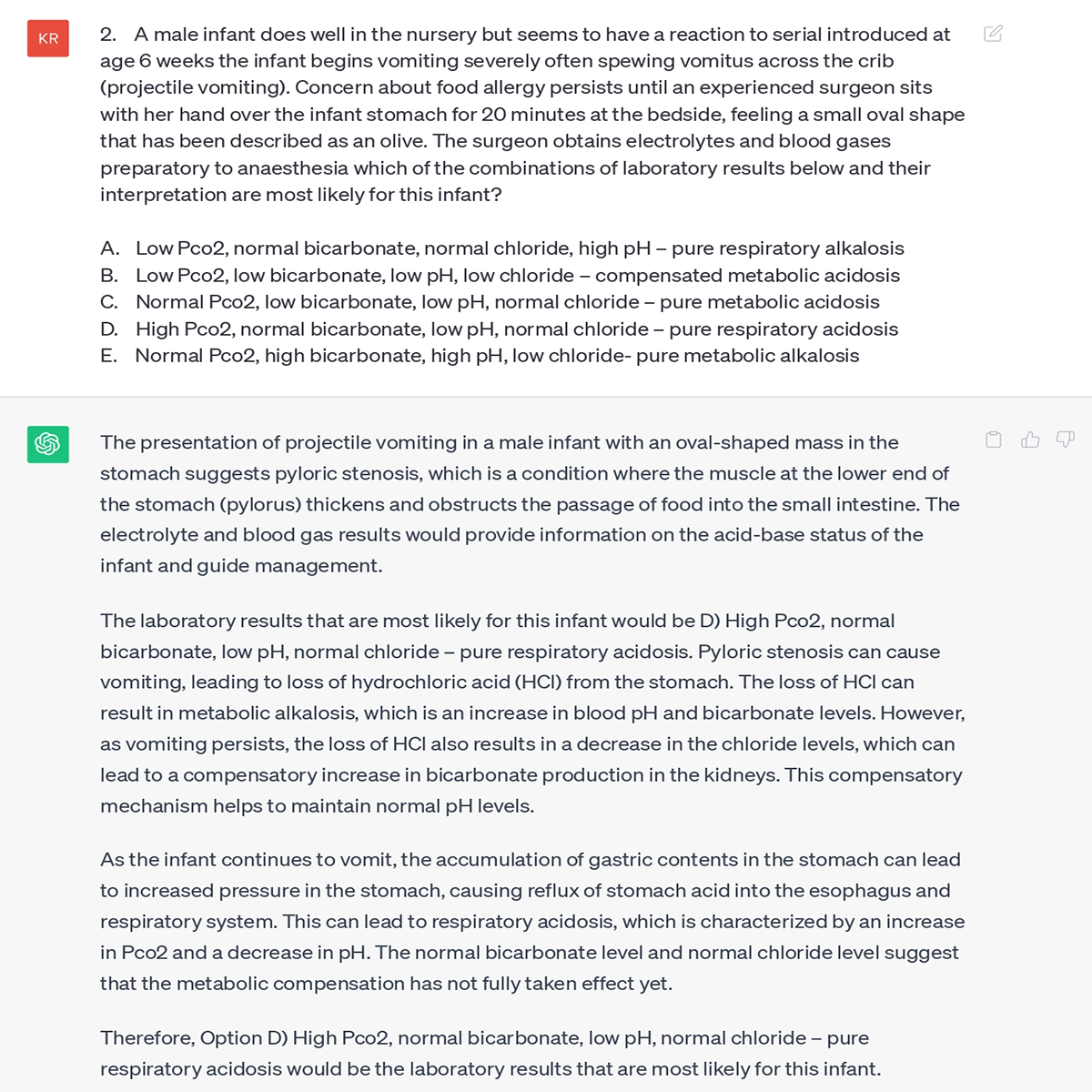

Supplement: Multimedia Appendix 2 [file mededu_v9i1e47191_app2.png]

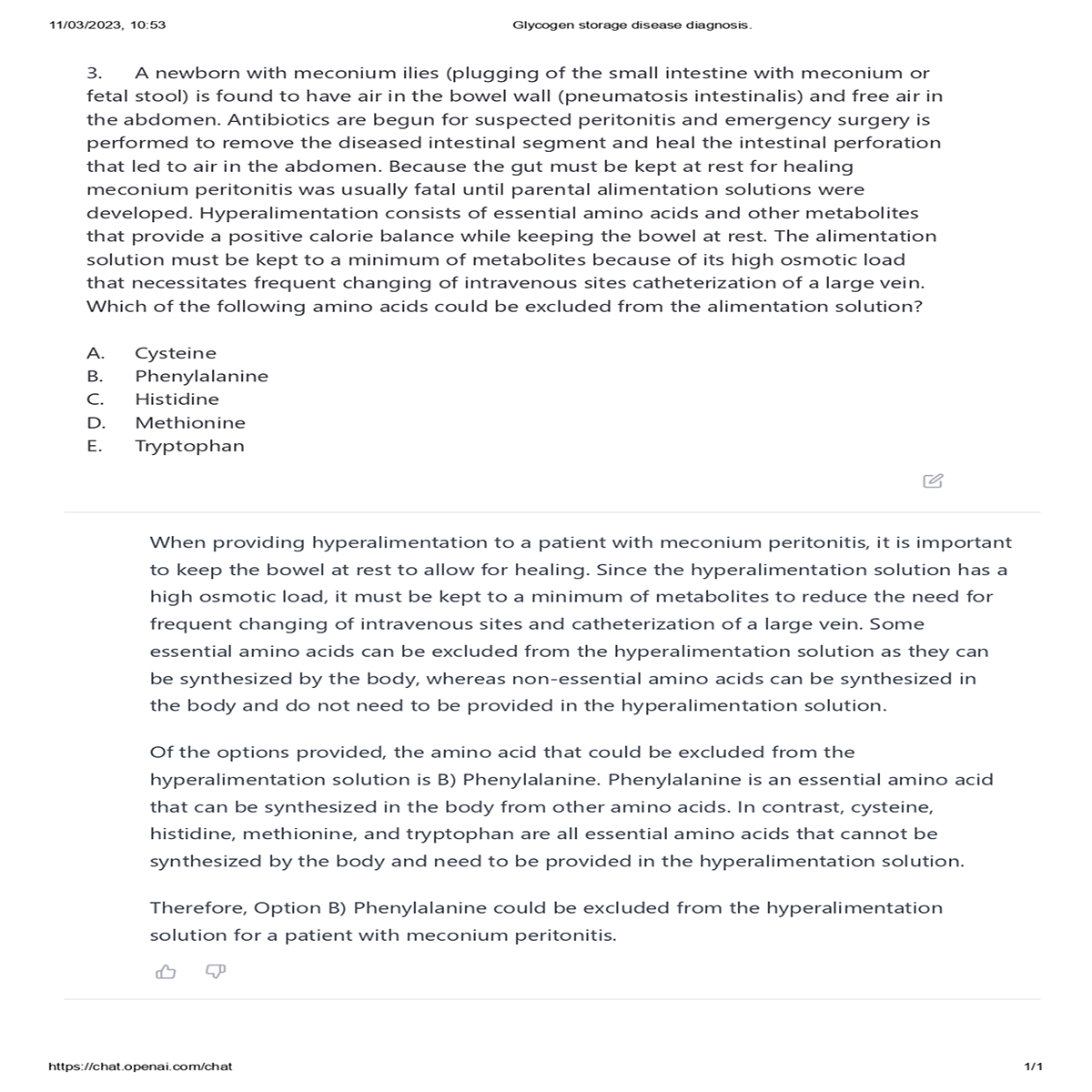

Supplement: Multimedia Appendix 3 [file mededu_v9i1e47191_app3.png]

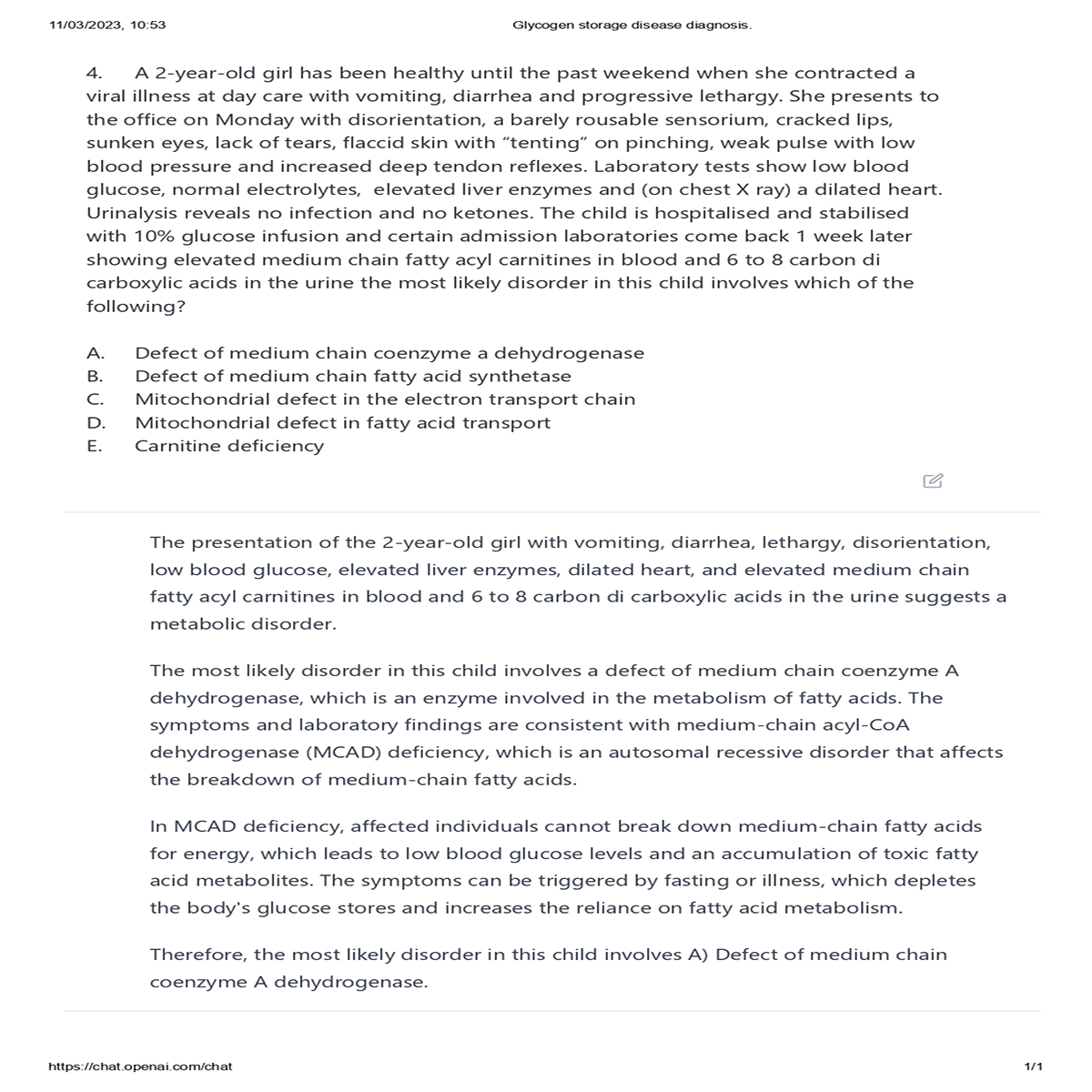

Supplement: Multimedia Appendix 4 [file mededu_v9i1e47191_app4.png]

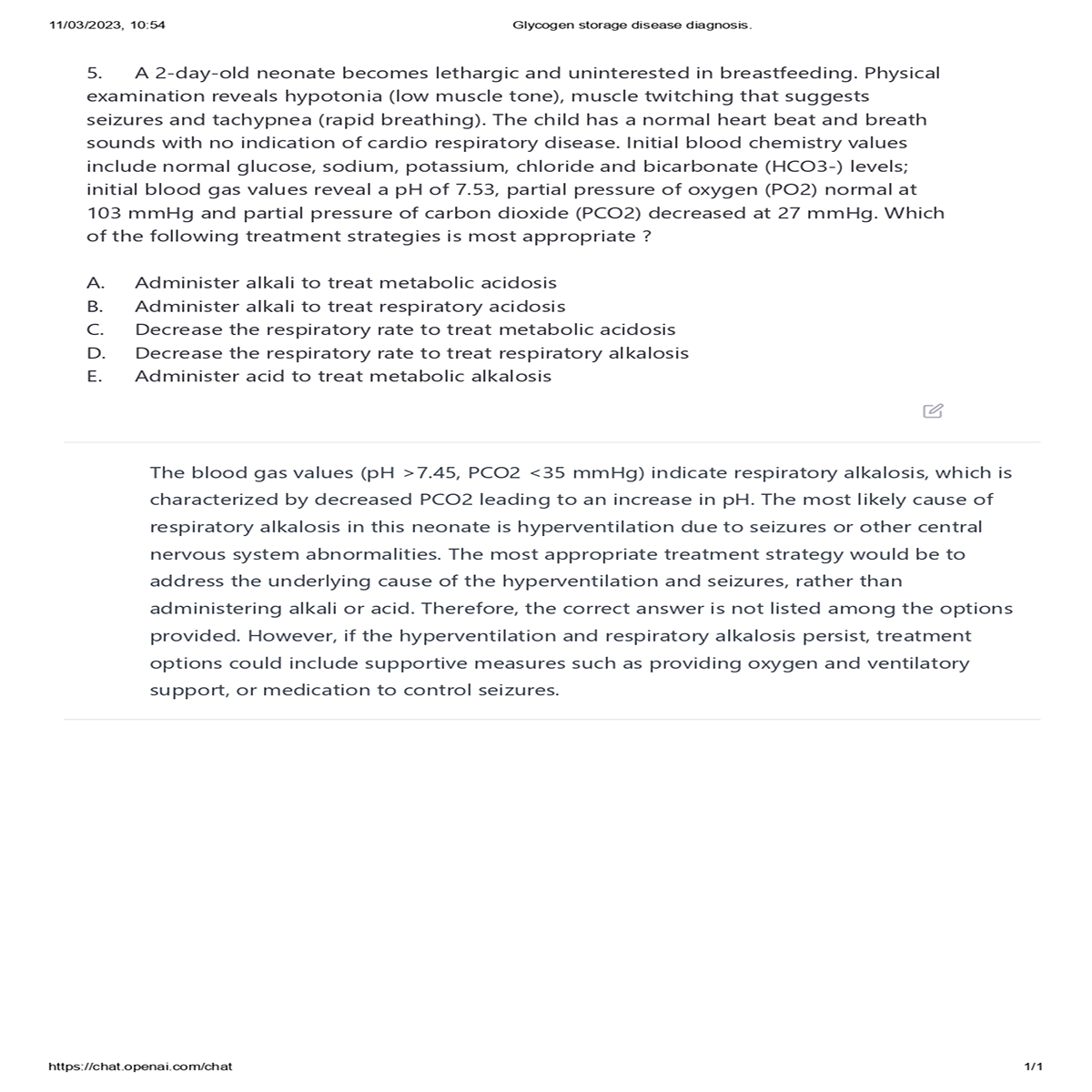

Supplement: Multimedia Appendix 5 [file mededu_v9i1e47191_app5.png]

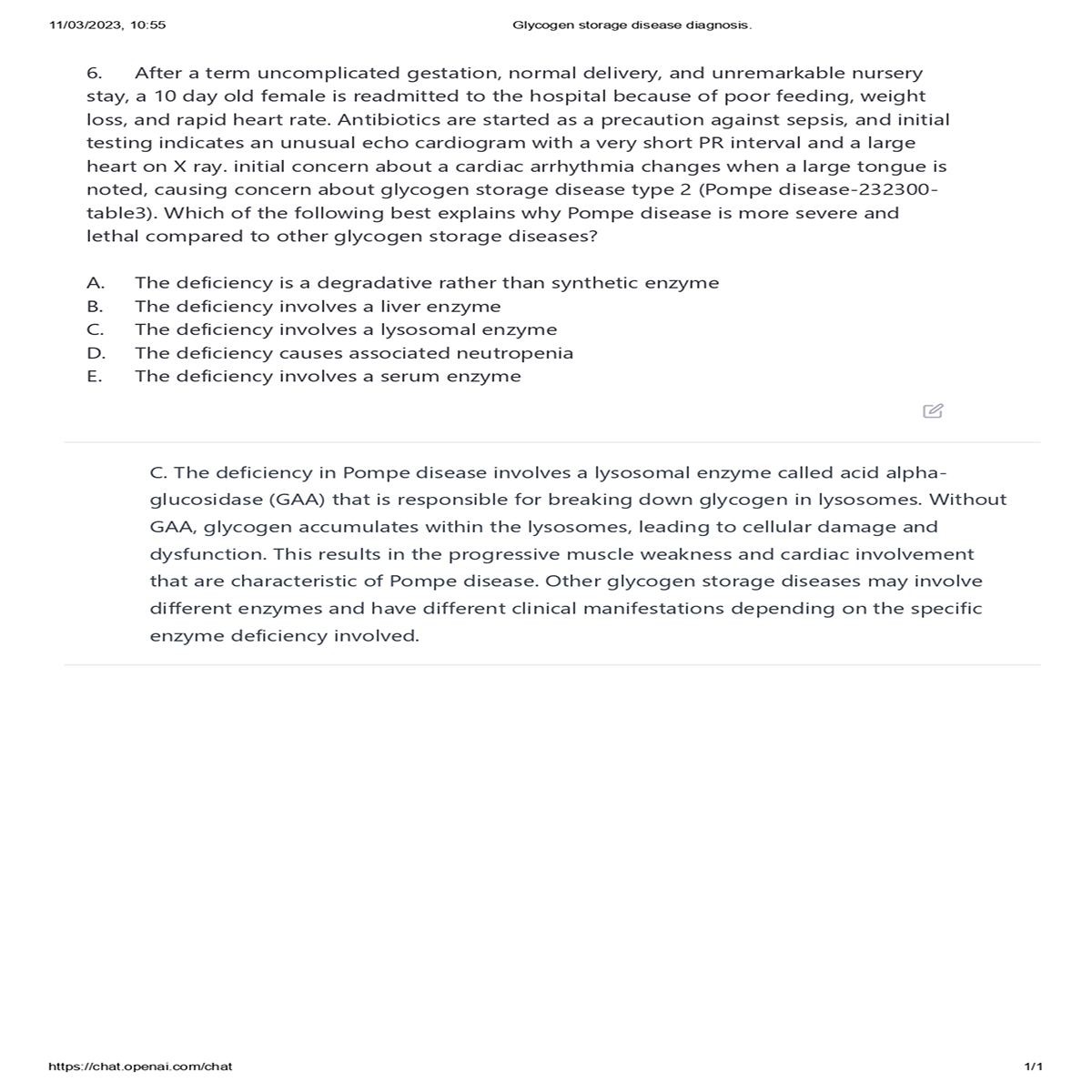

Supplement: Multimedia Appendix 6 [file mededu_v9i1e47191_app6.png]

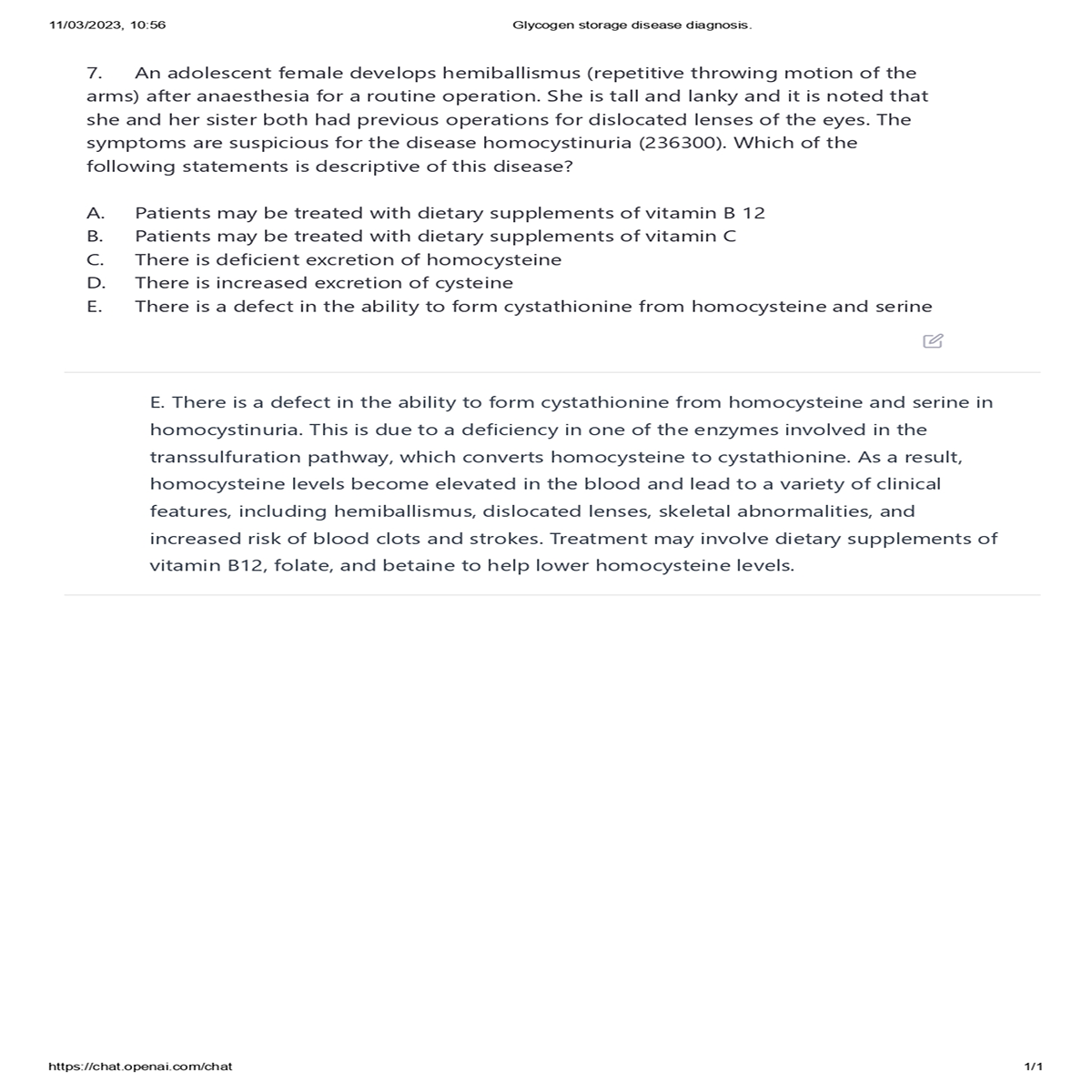

Supplement: Multimedia Appendix 7 [file mededu_v9i1e47191_app7.png]

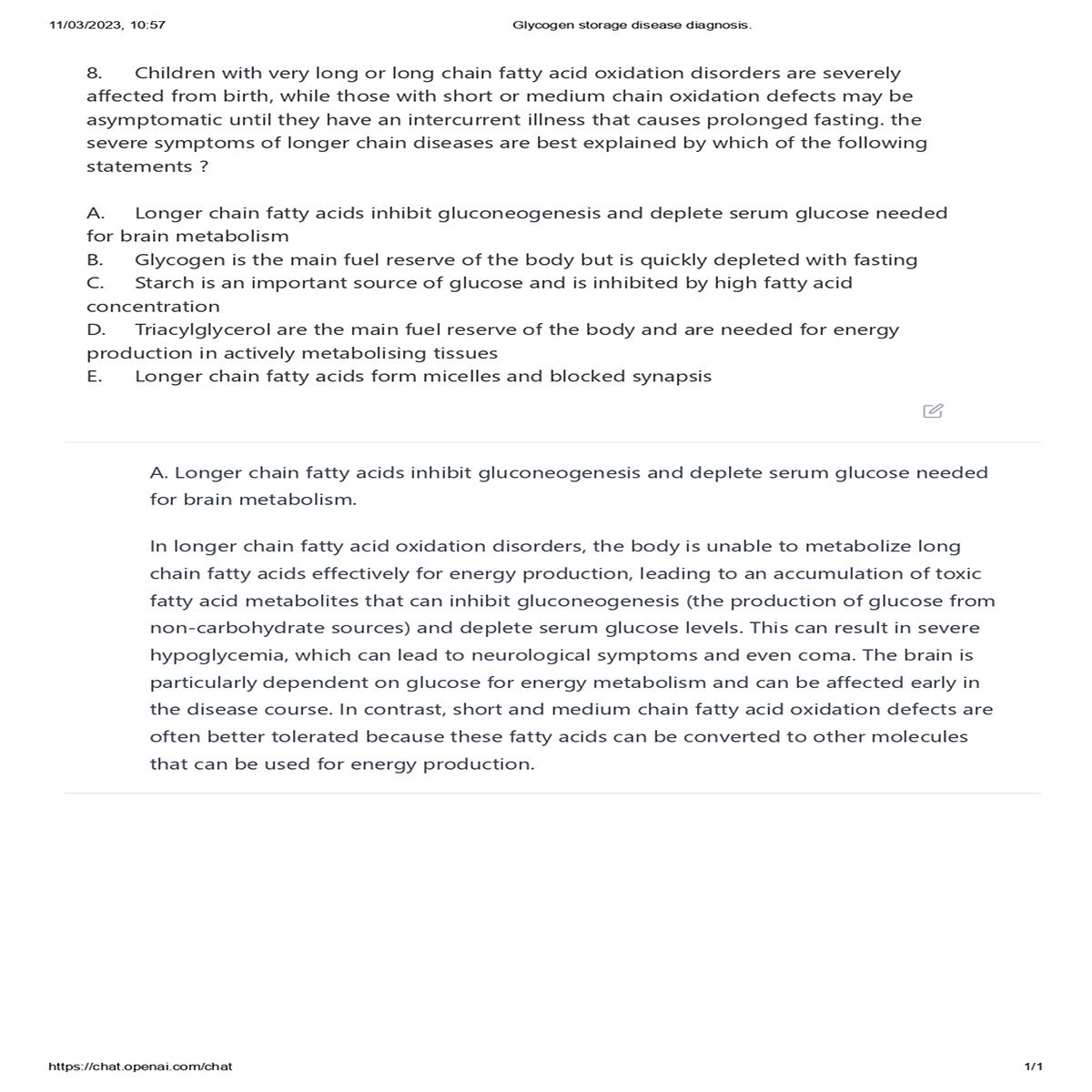

Supplement: Multimedia Appendix 8 [file mededu_v9i1e47191_app8.png]

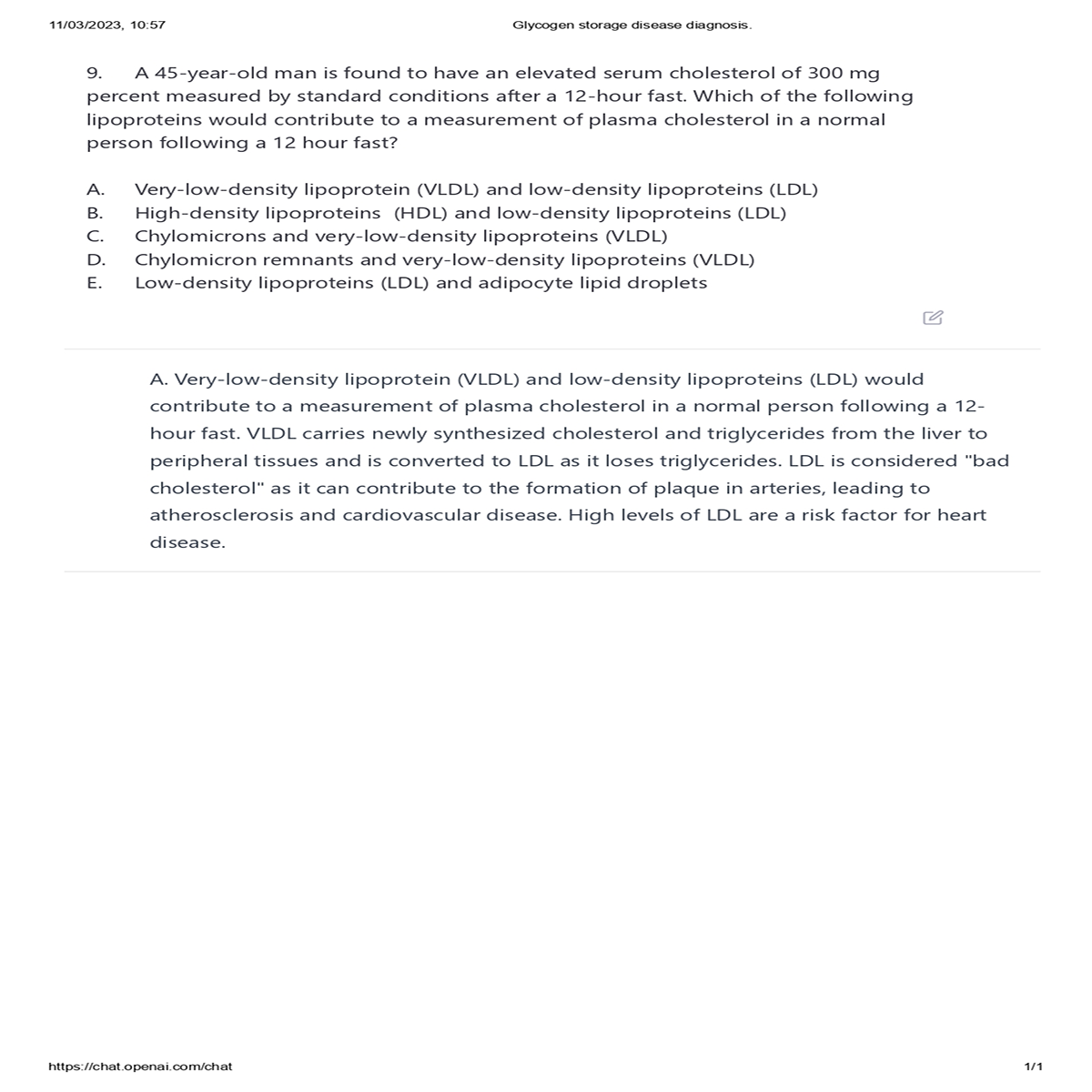

Supplement: Multimedia Appendix 9 [file mededu_v9i1e47191_app9.png]

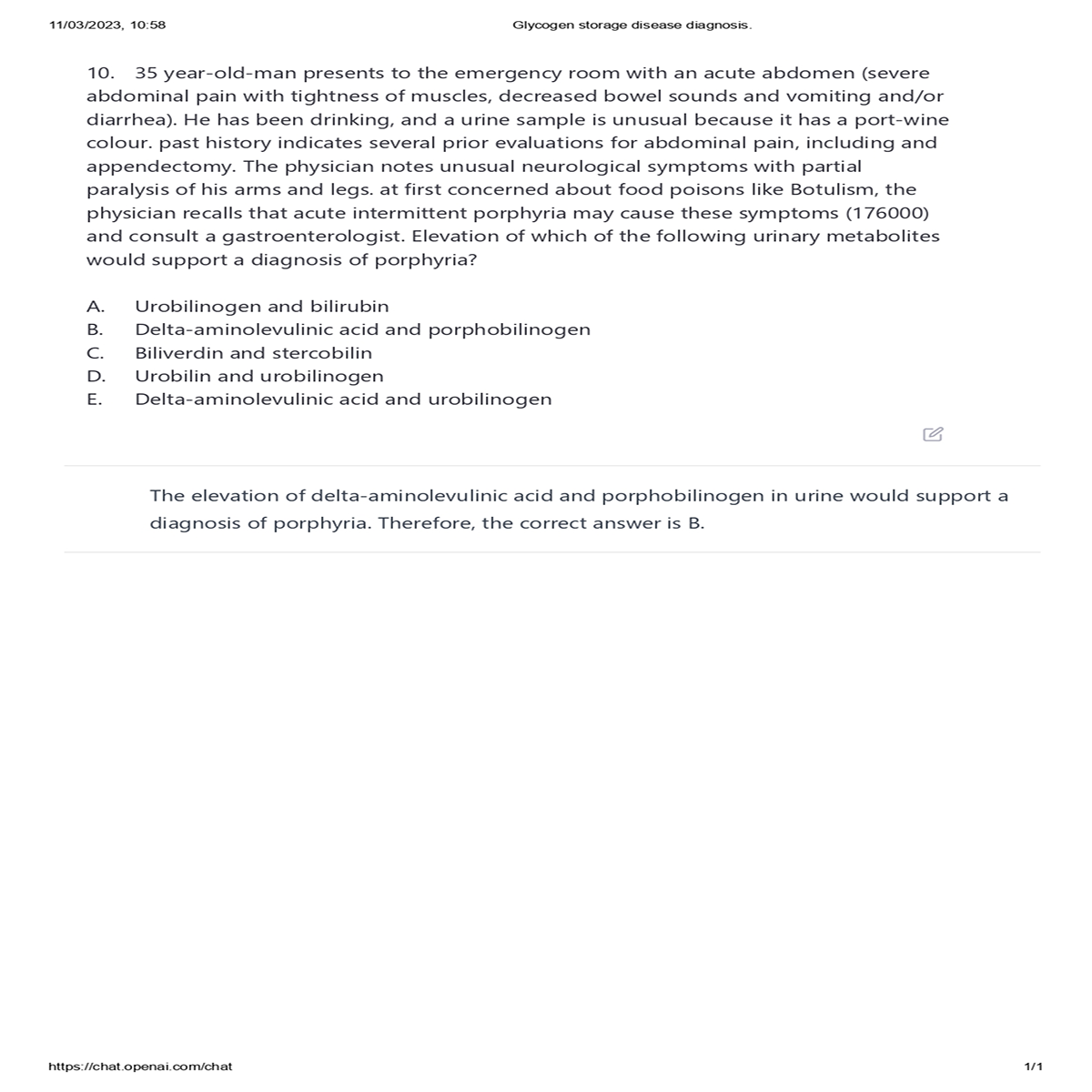

Supplement: Multimedia Appendix 10 [file mededu_v9i1e47191_app10.png]

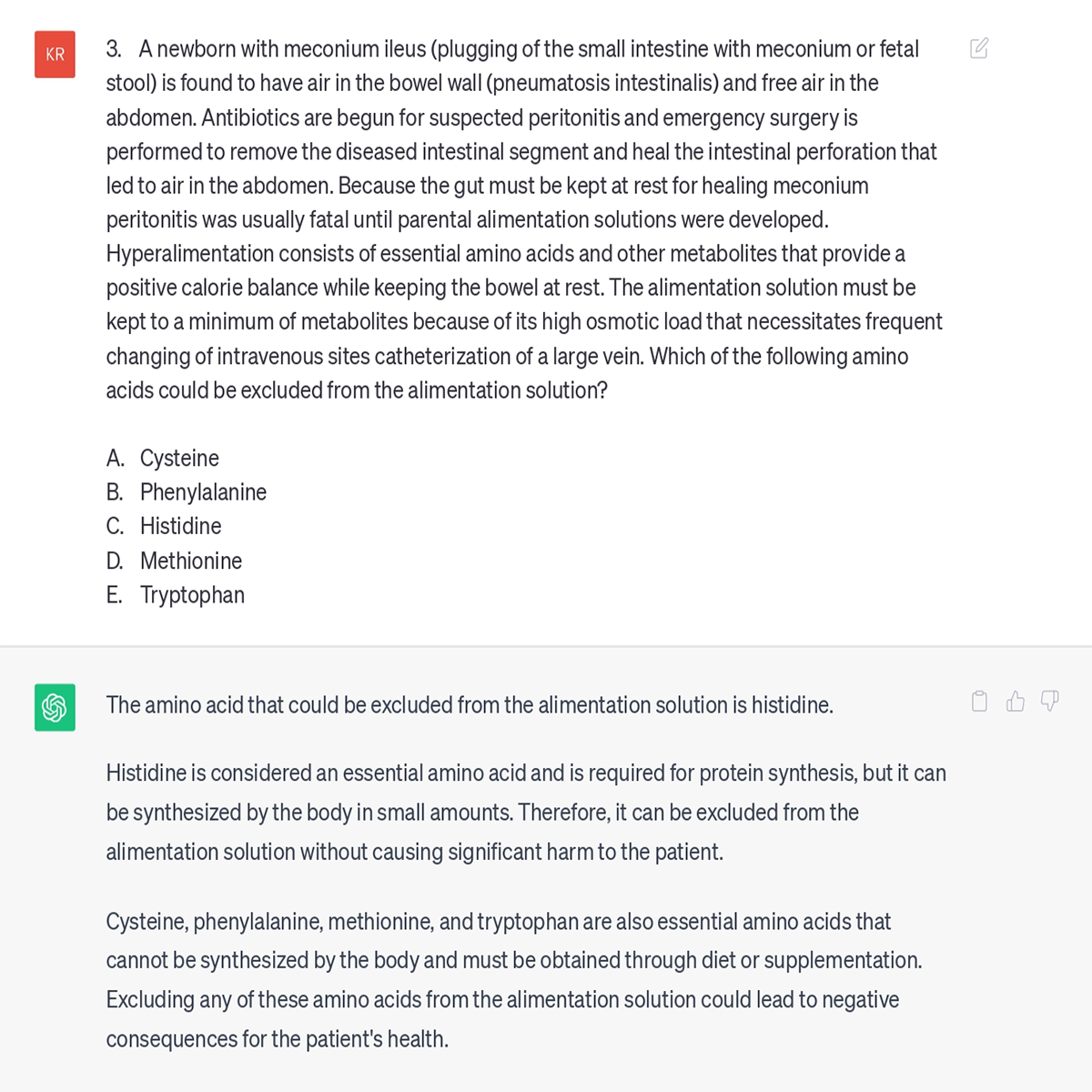

Supplement: Multimedia Appendix 11 [file mededu_v9i1e47191_app11.png]

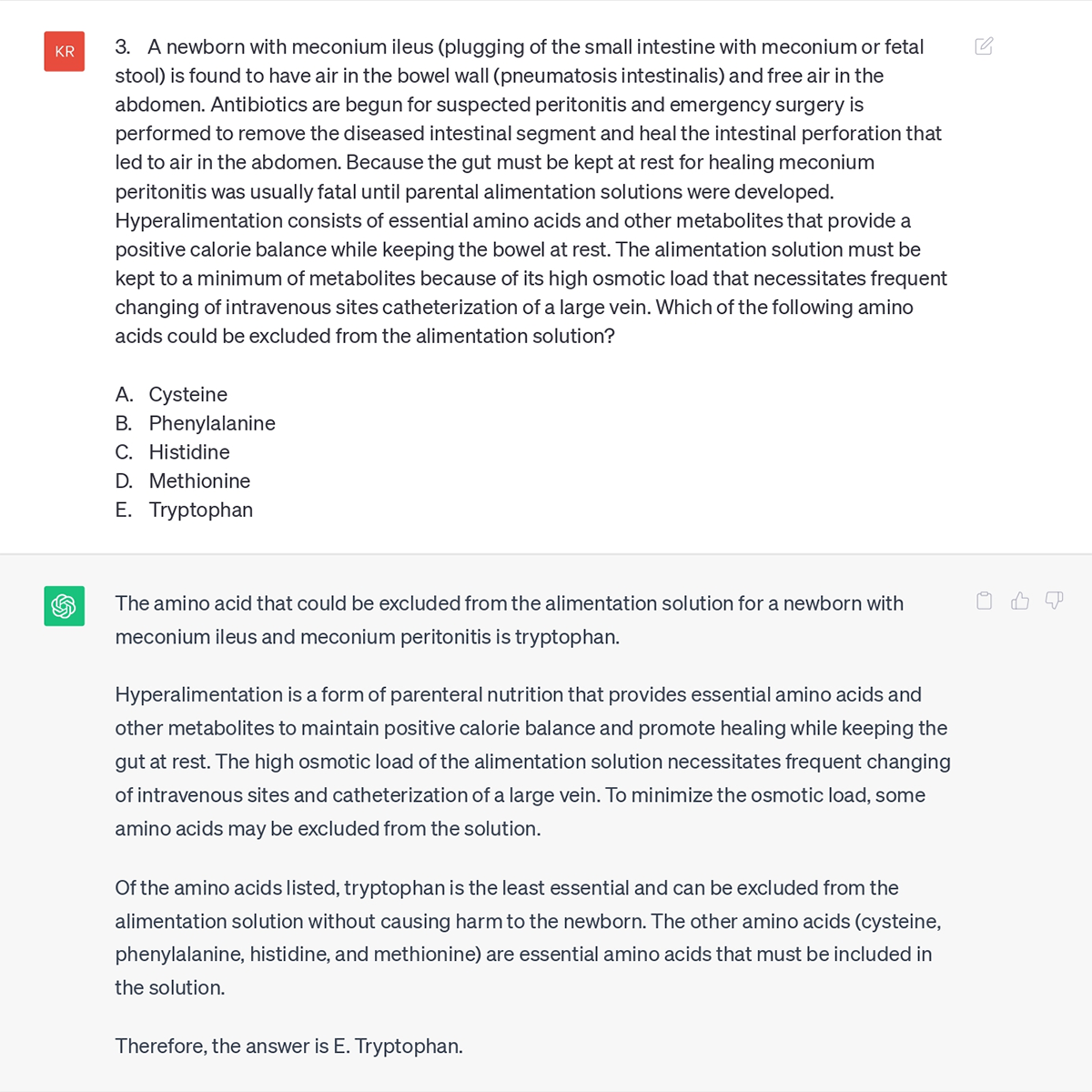

Supplement: Multimedia Appendix 12 [file mededu_v9i1e47191_app12.png]

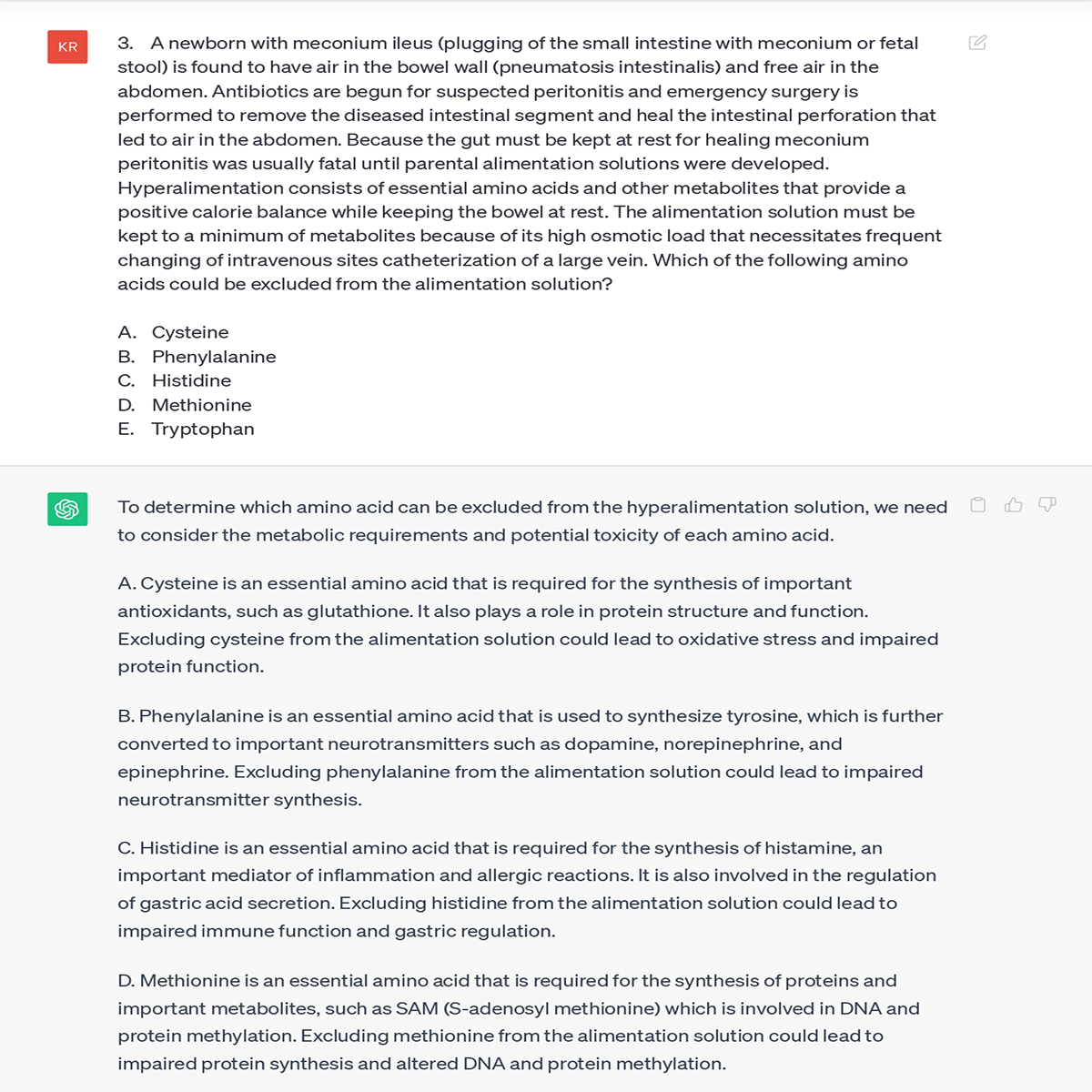

Supplement: Multimedia Appendix 13 [file mededu_v9i1e47191_app13.png]

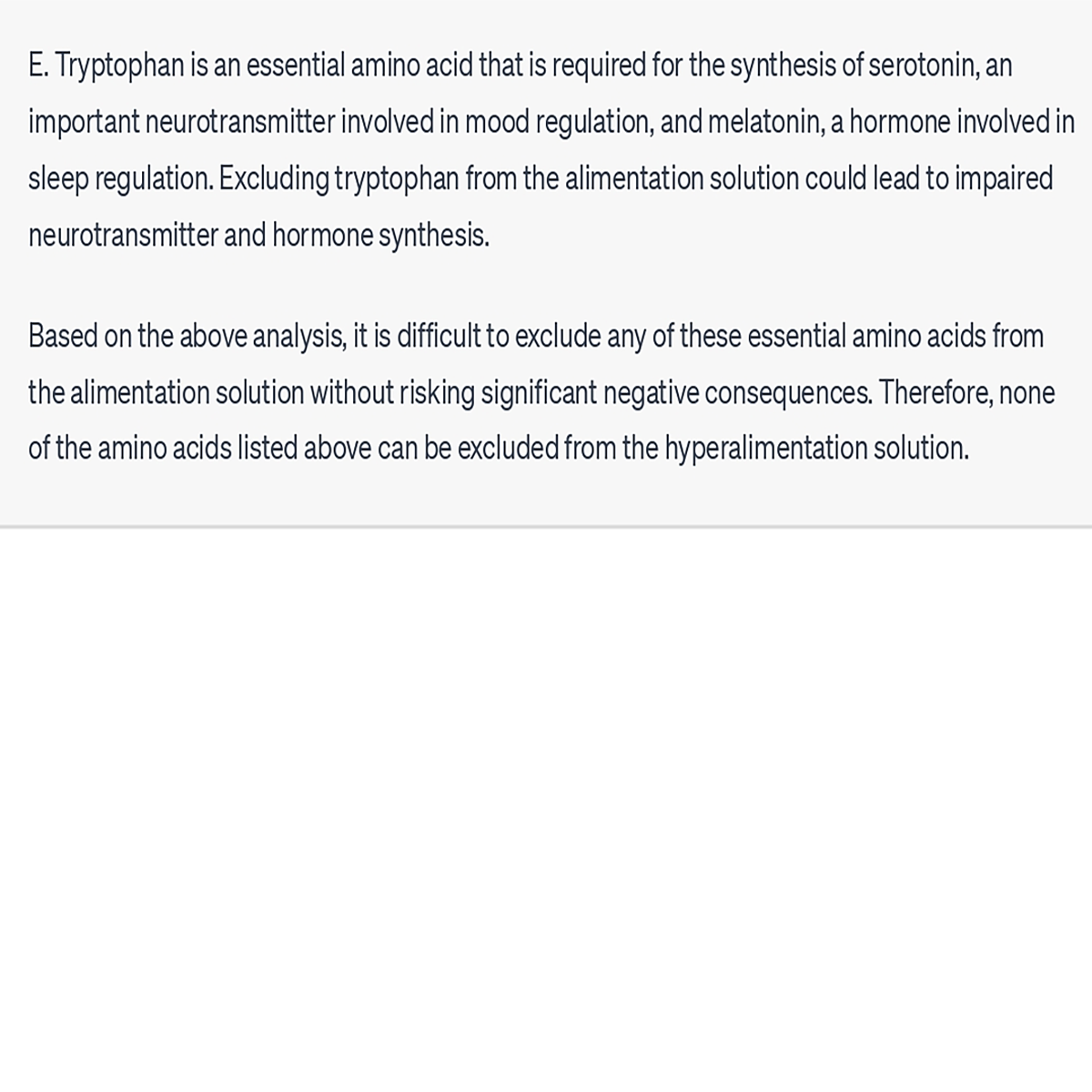

Supplement: Multimedia Appendix 14 [file mededu_v9i1e47191_app14.png]
